# Designing bifunctional perovskite catalysts for the oxygen reduction and evolution reactions†

**DOI:** 10.1039/d4ey00084f

**Published:** 2024-06-24

**Authors:** Casey E. Beall, Emiliana Fabbri, Adam H. Clark, Vivian Meier, Nur Sena Yüzbasi, Thomas Graule, Sayaka Takahashi, Yuto Shirase, Makoto Uchida, Thomas J. Schmidt

**Affiliations:** a Paul Scherrer Institute (PSI) 5232 Villigen PSI Switzerland emiliana.fabbri@psi.ch; b Institute for Physical Molecular Science, ETH Zürich 8093 Zürich Switzerland; c Empa 8600 Dübendorf Switzerland; d Hydrogen and Fuel Cell Nanomaterials Center, University of Yamanashi 400-0021 Kofu Japan

## Abstract

The development of unified regenerative fuel cells (URFCs) necessitates an active and stable bifunctional oxygen electrocatalyst. The unique challenge of possessing high activity for both the oxygen reduction (ORR) and oxygen evolution (OER) reactions, while maintaining stability over a wide potential window impedes the design of bifunctional oxygen electrocatalysts. Herein, two design strategies are explored to optimize their performance. The first incorporates active sites for the ORR and OER, Mn and Co, into a single perovskite structure, which is achieved with the perovskites Ba_0.5_Sr_0.5_Co_0.8_Mn_0.2_O_3−*δ*_ (BSCM) and La_0.5_Ba_0.25_Sr_0.25_Co_0.5_Mn_0.5_O_3−*δ*_ (LBSCM). The second combines an active ORR perovskite catalyst (La_0.4_Sr_0.6_MnO_3−*δ*_ (LSM)) with an OER active perovskite catalyst (Ba_0.5_Sr_0.5_Co_0.8_Fe_0.2_O_3−*δ*_ (BSCF)) in a physical mixed composite (BSCF/LSM). The success of the two strategies is investigated by measuring the catalysts’ catalytic performance and response to alternating reducing and oxidizing potentials to mimic the dynamic conditions experienced during the operation of URFCs. Additionally, the continuous, potentiodynamic change in Mn, Co, and Fe oxidation states during the ORR and OER is elucidated with *operando* X-ray absorption spectroscopy (XAS) measurements, revealing key insights into the nature of the active sites. The results reveal important catalyst physiochemical properties and provide a guide for future research and design principles for bifunctional oxygen electrocatalysts.

## Abstract 2

Design strategies for bifunctional catalysts are verified by investigating the catalysts’ activity and stability under reversible operation, as well as through *operando* investigation of the catalysts’ oxidation state.

## Graphical abstract

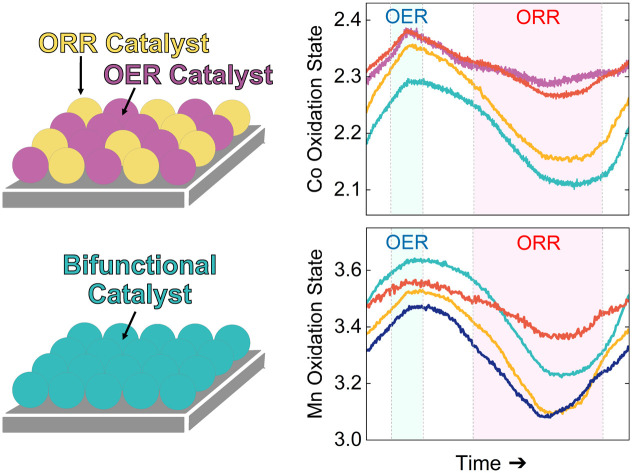

**Broader context**

Current climate issues require the replacement of fossil fuel-based technology with renewable energy systems. However, due to the irregular nature of renewable energy sources such as solar and wind, a flexible energy storage and conversion system is required. Hydrogen, with a high gravimetric energy density, is a favorable energy carrier. A system consisting of a water electrolyzer and hydrogen fuel cell can store and release energy from the bonds of hydrogen. A unified regenerative (or reversible) fuel cell (URFC) combines a fuel cell and water electrolyzer into one compact device, ideal for transportation applications and confined spaces. However, one of the main challenges hindering the advancement of URFCs is the activity and stability of the bifunctional oxygen electrode catalyst. This study investigates key design strategies that can be used to develop future bifunctional catalysts. The oxidation states of the catalysts are investigated *operando*, while alternating between fuel cell and electrolyzer operation, and the results demonstrate the challenge of balancing oxygen reduction and oxygen evolution activity in bifunctional catalysts.

## 1. Introduction

The demand for clean, renewable energy, due to the imminent global climate crisis, has stimulated the search for energy conversion and storage devices.^1,2^ A hydrogen-based energy system is promising due to its zero emissions and the high gravimetric energy density of hydrogen.^3,4^ Unified regenerative fuel cells (URFCs) combine a hydrogen fuel cell with a water electrolyzer into one device. Therefore, they have the unique possibility of requiring less materials, and lower cost, weight, and volume than a combined fuel cell and electrolyzer system.^5,6^ These properties make URFCs particularly valuable for remote areas, transportation and aerospace applications, and confined spaces.^5,7^ The reversible fuel cells switch between operating as either a fuel cell, to release energy, or an electrolyzer, to store energy in hydrogen. The oxygen reduction reaction (ORR) and oxygen evolution reaction (OER), which occur at the oxygen electrode during fuel cell and electrolyzer operation, respectively, suffer from sluggish kinetics and high overpotentials, leading to efficiency losses.^8,9^ Additionally, bifunctional catalysts have the unique challenge of retaining performance and stability over a wide potential range from oxidizing to reducing potentials, as well as of withstanding alternating operation between these two extreme conditions. Alternating conditions have proven to be detrimental to the longevity and performance of a variety of bifunctional catalysts, although they demonstrate sufficient activity for both the ORR and OER.^10–14^ Presently the search for an active and stable bifunctional oxygen electrocatalyst remains one of the key hindrances to URFC development.^13,15,16^

Perovskite-type oxides (A_1−*x*_A′_*x*_B_1−*y*_B′_*y*_O_3−*δ*_) are an important class of materials for oxygen electrocatalysis in alkaline electrolyte.^17–26^ Their low cost and adaptability^20,27,28^ allow for the careful design of catalysts active for both the ORR and OER. Perovskites have been shown previously to be promising bifunctional catalysts.^18,29–34^ The catalytic active sites for the ORR and OER in perovskites have been hypothesized to be the B-site metals.^19,20,35–37^ However, an active site for the ORR is not inherently active for the OER. Indeed, the catalytic properties and compositions that lead to high activity for the ORR or OER are divergent.^32,38^ Accordingly, performance optimization for bifunctional catalysts is challenging and conscientious design strategies are required. This study aims to investigate different methods for designing bifunctional oxygen electrocatalysts in order to guide future studies.

Herein, two strategies of bifunctional catalyst design are described and compared. The first strategy for bifunctional catalyst design is to create a single-phase material containing active sites for both the ORR and OER. The selected active sites are Mn and Co, which have been shown to catalyze the ORR and OER.^39–42^ The single-phase perovskites Ba_0.5_Sr_0.5_Co_0.8_Mn_0.2_O_3−*δ*_ (BSCM) and La_0.5_Ba_0.25_Sr_0.25_Co_0.5_Mn_0.5_O_3−*δ*_ (LBSCM) were designed in this study with inspiration from two well-known perovskites, Ba_0.5_Sr_0.5_Co_0.8_Fe_0.2_O_3−*δ*_ (BSCF) with high OER activity^18,43,44^ and La_*x*_Sr_1−*x*_MnO_3−*δ*_ (LSM) with high ORR activity.^45–47^ In the Ba_0.5_Sr_0.5_Co_0.8_Mn_0.2_O_3−*δ*_ (BSCM) perovskite structure, Fe in BSCF is replaced with Mn with the purpose of increasing its ORR activity. Furthermore, the perovskite La_0.5_Ba_0.25_Sr_0.25_Co_0.5_Mn_0.5_O_3−*δ*_ (LBSCM) was developed, which combines 50% Co with 50% Mn at the B-site to have active sites for both reactions.

The second strategy is to create a composite, which combines an active ORR catalyst with an active OER catalyst. This strategy allows for the optimization of one catalyst for the ORR and the other for the OER. In contrast, the single-phase strategy must always compromise between properties leading to higher activity for one reaction over the other. Various methods can be used to create composites such as a layered structure, a homogeneous mixture, or a core–shell design.^10–12,48–51^ In this study, a homogeneous mixture is created by combining two active perovskites in a 1 : 1 weight ratio. BSCF is chosen as the active OER catalyst and LSM as the active ORR catalyst to create the composite BSCF/LSM while additionally serving as good comparison to the single-phase materials BSCM and LBSCM. Previous composites of BSCF and LSM include a layered epitaxial thin film of BSCF on (001)-orientated LSM^48^ for low temperature applications, a core (BSCF) shell (LSM) structure,^52^ and BSCF infiltrated with LSM^53^ for solid oxide fuel cells.

As URFC operation dynamically switches between fuel cell and electrolyzer operation, the study performed here is designed to mimic the alternating reducing and oxidizing potentials in order to assess the catalyst's bifunctional performance. Additionally, the effects of OER operating conditions on subsequent ORR performance and *vice versa* are investigated. *Operando* X-ray absorption spectroscopy (XAS) is utilized to follow the potentiodynamic changes in Mn, Co, and Fe oxidation states while performing cyclic voltammetry between reducing and oxidizing potentials. In this way, the changes in catalyst behavior and performance degradation are clarified. Then, evaluation of device level performance is assessed using anion exchange membrane (AEM) fuel cell and water electrolyzer measurements, showing significant variation from rotating disk electrode (RDE) measurements. With these methods, the feasibility of these catalysts is rigorously tested and a thorough comparison of the two design strategies is achieved. Finally, the results of this study are used to give recommendations towards the rational design of bifunctional oxygen electrocatalysts.

## 2. Experimental methods

### 2.1. Material synthesis

Perovskite nanoparticles were synthesized using the flame spray method described previously.^54,55^ The precursor solution of acetic acid (≥99.0%, Roth) and milliQ water contained stoichiometric amounts of the metal nitrates for a total metal concentration of 0.1 M (lanthanum nitrate tetrahydrate (99.9%, Sigma-Aldrich), barium carbonate (≥99.0%, Sigma-Aldrich), strontium nitrate (≥98%, Sigma-Aldrich), cobalt nitrate hexahydrate (99.9%, Sigma-Aldrich), manganese nitrate tetrahydrate (≥97%, Sigma-Aldrich), and iron nitrate nonahydrate (≥98%, Sigma-Aldrich)). This solution had a flow rate of 10 or 50 mL min^−1^ through the flame. The combustion gas mixture (acetylene (99.6%, Carbagas) and oxygen (99.5%, Carbagas)) flow rate was 13 or 15 L min^−1^ and the dispersion gas (oxygen) was 25 L min^−1^. Acetylene carbon black (>99.9%, Alfa Aesar) was bought commercially. The Co, Fe, and Mn references for XAS were bought commercially [(Co(OH)_2_, ≥99.9%, Thermo Scientific); (Fe_2_O_3_, ≥99.995%, Aldrich); (Fe_3_O_4_, 99.999%, Sigma-Aldrich); (MnO, 99.99%, Sigma-Aldrich); (Mn_2_O_3_, 98%, Alfa Aesar); (MnO_2_, >99%, Sigma-Aldrich)].

### 2.2. *Operando* XAS

X-ray absorption spectroscopy (XAS) measurements were completed at the SuperXAS beamline at the Swiss Light Source (PSI, Switzerland). The photon beam source was a 2.9 T superbend magnet collimated with a Si coated mirror at 2.8 mrad and monochromatized with channel cut Si (111). A double focusing mirror coated with Rh was used to focus the beam (0.2 × 1 mm^2^). The sample was located between the first and second chambers and measured in quick fluorescence mode^56^ (using a PIPS detector). The reference metal foil (Co at the Co and Fe K-edges or Fe at the Mn K-edge) was located between the second and third chambers and measured in transmission. Oxidation state references were measured in transmission as *ex situ* pellets. The software ProQEXAFS was used for data analysis, energy calibration, and normalization.^57^ A homemade flow cell described previously^58^ was used for *operando* measurements. The working electrode consisted of gold sputtered carbon coated Kapton sprayed with catalyst ink (isopropanol, milliQ water, and Na^+^-exchanged Nafion, sonicated 1 h). The counter electrode was sprayed with black pearl (2000 carbon black, Cabot Corporation). The reference electrode was Ag/AgCl (3 M NaCl filled, Harvard Apparatus). A syringe pump was used to flow oxygen-saturated 0.1 M KOH through the flow cell at a rate of 0.4 mL min^−1^. The initial oxidation states of the dry catalyst samples are shown in Table S4 (ESI†) and the oxidation calculations are elucidated in Note S1 (ESI†).

### 2.3. Electrochemical measurements

The catalysts were evaluated using thin-film rotating disk electrode (RDE) methodology.^59,60^ All RDE measurements were conducted in oxygen-saturated 0.1 M KOH (99.99%, Sigma Aldrich) with a rotation rate of 1600 rpm and a scan rate of 5 mV s^−1^, unless otherwise stated. The measured current was normalized by the area of the glassy carbon electrode surface (0.196 cm^2^). The Hg/HgO reference electrode (0.1 M KOH filled, RE 61AP, ALS) was converted to RHE (0.925 V) by calibrating against Pt mesh in hydrogen saturated electrolyte. All potentials are given *versus* RHE and ohmic drop corrected using impedance spectroscopy. Gold mesh was used as the counter electrode. The working electrode consisted of 15 μL of catalyst ink (perovskite (10 mg), acetylene carbon black (2.8 mg), milliQ water (1.5 mL), isopropanol (1 mL), and Na^+^-exchanged Nafion (20 μL) (5 wt%, Sigma Aldrich)) dropcasted onto the surface of a polished glassy carbon disk. The ORR activity of the bare glassy carbon was measured before every measurement as a reference. The ink was sonicated for 30 min.

For AEM evaluation, an ammonium containing copolymer (QPAF-4) described previously^61^ was used for the membrane and binder. The catalyst inks had a X (FC) and X (WE) catalyst/binder ratio and were sprayed using a pulse-swirl technique (PSS, Nordson Co. Ltd). The perovskite inks were sprayed onto the membrane and the CCMs had an area of 4.41 cm^2^ (FC) and 1 cm^2^ (WE). BSCF/LSM/carbon contained 22 wt% carbon, 39 wt% BSCF, and 39 wt% LSM. For AEMFC the cell temperature was 60 °C and the feeding gases (100 mL min^−1^) were humidified to 100% RH. On the anode, Pt/CB CCM and carbon cloth with MPL (W1S1010, CeTech) GDL were used and on the cathode the GDL was carbon paper with MPL (22BB, Sigracet). The cell was compressed to a pressure 10 kgf cm^−2^. The AEMFC set-up was explained previously.^62^ The current density was increased stepwise until 1.0 A cm^−2^ and held at each step for one minute. For AEMWE, the cell was operated at a temperature of 80 °C and compressed to a pressure of 0.75 MPa. The 1 M KOH electrolyte was preheated to 80 °C and was recirculated with a flow rate of 10 mL min^−1^. The cathode GDE consisted of Pt/CB (46.9% Pt, TEC10E50E, Tanaka) sprayed onto Teflon treated carbon fiber paper (TGP-H-120, Toray). The MEA was pressed at 0.2 kN and 80 °C. The current density was increased twice to 1.0 A cm^−2^ and twice to 2 A cm^−2^ and held at each step for 30 s. Further experimental details are described in the ESI.†

## 3. Results and discussion

**Fig. 1:**
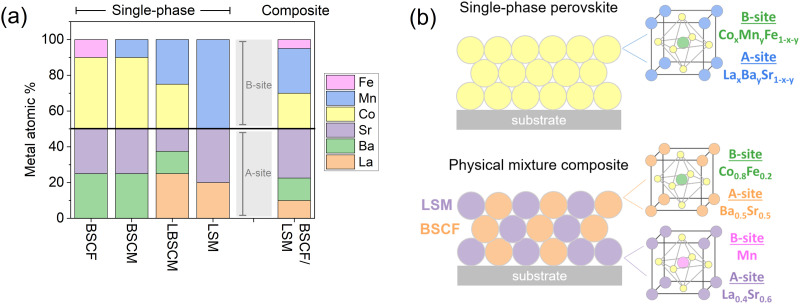
(a) The metal concentration of the perovskites studied in this work. Oxygen is not included in the percentage. The elements La, Ba, and Sr occupy the A-site of the perovskite structure (ABO_3_) and the elements Co, Mn, and Fe occupy the B-site. (b) Schematic diagram of the catalyst layer for the two design strategies, a single-phase material and a physical mixture composite. The composition is calculated from synthesis stoichiometry.

The perovskites investigated in this study contain La, Ba, and/or Sr on the A-site of the crystal structure (ABO_3_) and Mn, Co, and/or Fe on the B-site. The composition of the materials by metal atomic percentage is elaborated in Fig. 1a. The mixed composite BSCF/LSM contains a physical mixture of 50% BSCF and 50% LSM by mass. A homogeneous mixture was obtained by adding BSCF and LSM, with a 1 : 1 weight ratio, into an ink and sonicating them. The ink was then dropcast onto the electrode surface, creating a physical mixture of the two perovskites in the catalyst layer. The composition of the catalyst layer is represented visually in Fig. 1b.

X-ray diffraction (XRD) measurements show the flame spray synthesized materials crystalize into cubic perovskite structures with some secondary phases identified as carbonates and nitrates (Fig. S1, ESI†). The conductivities of the nanopowder perovskite samples were measured *via ex situ* 4-wire impedance spectroscopy (Table S2, ESI†). The overall conductivity (bulk/grain contact) decreases in the order: LBSCM > LSM > BSCM > BSCF. Thus, carbon was added to the catalyst layer to equalize electrical conductivity within the different catalyst layers (Table S3, ESI†). Previously, the influence of carbon on the ORR activity of perovskites and the change in Co oxidation state has been investigated and shown to enhance Co reduction during the ORR, as well as Co oxidation during the zOER, and correlated with increased catalytic activity for both reactions.^19,20^

### 3.1. Catalytic activity

The activity of the catalysts for the ORR and OER was investigated using the thin-film rotating disk electrode (RDE) methodology^59,60^ in 0.1 M KOH. In the ORR polarization curves, the current plateau characteristic of a diffusion-controlled reaction regime was not observed for any of the investigated samples, indicating that the ORR is under mixed kinetic-diffusion control over the entire potential range. In addition, each sample exhibits a two-wave ORR curve, most likely due to the presence of carbon in the electrodes; indeed, the two-wave ORR curve is typical of carbon electrodes in alkaline media.^63–65^

**Fig. 2:**
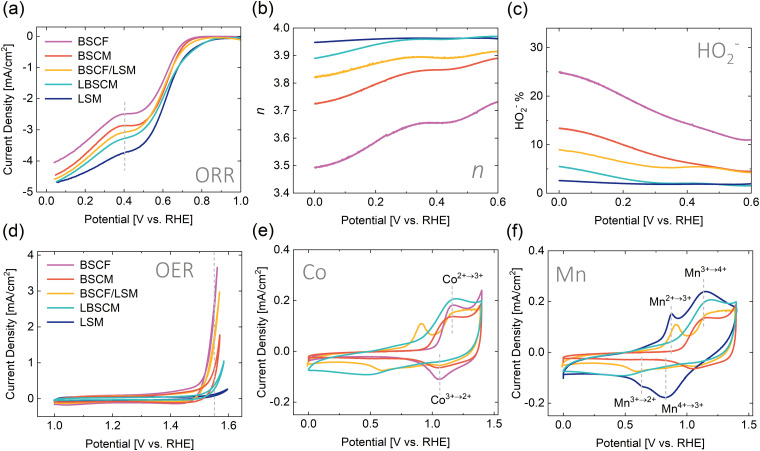
(a) The ORR polarization curves in oxygen saturated electrolyte. (b) The number of electrons transferred during the ORR (*n*) and (c) the hydrogen peroxide yield measured using RRDE. The Pt-ring was held at 1.2 V_RHE_, where the detection of peroxide species is diffusion limited. (d) OER polarization curves in oxygen saturated electrolyte. Cyclic voltammograms in argon saturated electrolyte showing the redox activity of the (e) Co containing and (f) Mn containing perovskite catalysts. The redox peaks are indicated by dotted grey lines in (e) and (f). The dotted grey lines in (a) and (d) indicate the potential at which the catalytic activity can be estimated. All measurements were conducted in 0.1 M KOH with a rotation rate of 1600 rpm and a scan rate of 5 mV s^−1^. For all graphs (a)–(f) the colors always correspond to the same samples: purple – BSCF, maroon – BSCM, yellow – BSCF/LSM, turquoise – LBSCM, and blue – LSM.

Interestingly, the trend in ORR activity, determined as the value of the geometric current density at an applied potential of 04. V *vs.* RHE, follows the percentage of Mn occupying the B-site (LSM > LBSCM > BSCF/LSM > BSCM > BSCF) (Fig. 1a and 2a). When Co is coupled with Mn in LBSCM, the ORR activity slightly decreases compared to LSM; however, the onset is the same. The substitution of Fe with Mn(BSCM) leads to higher ORR activity compared to BSCF. The physical mixture BSCF/LSM presents an intermediate ORR activity between LSM and BSCF. Additionally, the percentage of Mn influences the ORR reaction mechanism, as evidenced by the rotating ring disk electrode (RRDE) methodology in Fig. 2b. A 4-electron process is preferred for the ORR because a higher energy conversion efficiency is achieved and potentially harmful intermediate peroxide species are removed. The number of electrons transferred during the ORR increases significantly with the addition of Mn into BSCF, both through substitution (BSCM) and the physical mixture with LSM (BSCF/LSM). This would indicate the high ability of Mn to react with hydrogen peroxide, as evidenced by the results in Fig. 2c, even when only present at 20% on the B-site and regardless of Mn's position in the catalyst layer, either in a composite or single-phase electrode. Indeed, Mn-based oxides have been shown previously to catalyze the reaction of hydrogen peroxide and are used for hydrogen peroxide decomposition applications.^66–68^

In contrast, the OER activity of the materials, described here as the current density recorded during the anodic scan at the applied potential of 1.55 V RHE, (BSCF > BSCF/LSM > BSCM > LBSCM > LSM) roughly follows the percentage of Co at the B-site in Fig. 2d. Interestingly, the composite BSCF/LSM is surprisingly active considering it only contains 50 wt% BSCF. In separate loading experiments (Fig. S3, ESI†), it is revealed that BSCF/LSM has higher OER activity than the sum of its counterparts indicating a synergistic relationship between the two materials. Although BSCM contains the same ratio of Co as BSCF, with the loss of Fe, BSCM experiences a significant drop in OER activity. Fe doping has been shown to have a positive effect on the oxygen evolution activity and stability for Co containing materials.^69,70^ However, our attempt to produce Fe doped BSCM with flame spray synthesis failed to produce the correct phase (Fig. S4, ESI†).

The redox activity of the materials can be observed using cyclic voltammetry (CV) in Ar-saturated electrolyte. Several redox peaks can be seen in Fig. 2e and f potentially ascribed to the Mn(ii/iii), Mn(iii/iv), and Co(ii/iii) redox peaks, indicating the materials’ ability to change oxidation state within the potential range of interest. For LSM, the Mn(ii/iii) and Mn(iii/iv) transitions can be observed around 0.75 V and 1.0 V *vs.* RHE, respectively.^71^ For BSCF, the Co(ii/iii) transition is apparent around 1.1 V.^72^ The Co(ii/iii) and Mn(iii/iv) redox peaks overlap and therefore, for the other materials which contain both Co and Mn, it is difficult to distinguish these transitions. The BSCF/LSM composite exhibits the redox peaks of both BSCF and LSM. For the single materials BSCM and LBSCM, the redox peaks are not as defined but still apparent. With the RDE results shown so far, BSCF/LSM is the material most adept at combining OER and ORR activity. Although the single materials containing both Co and Mn, BSCM and LBSCM, have good ORR performance, their OER activity is quite poor.

### 3.2. Response to alternating operation

**Fig. 3:**
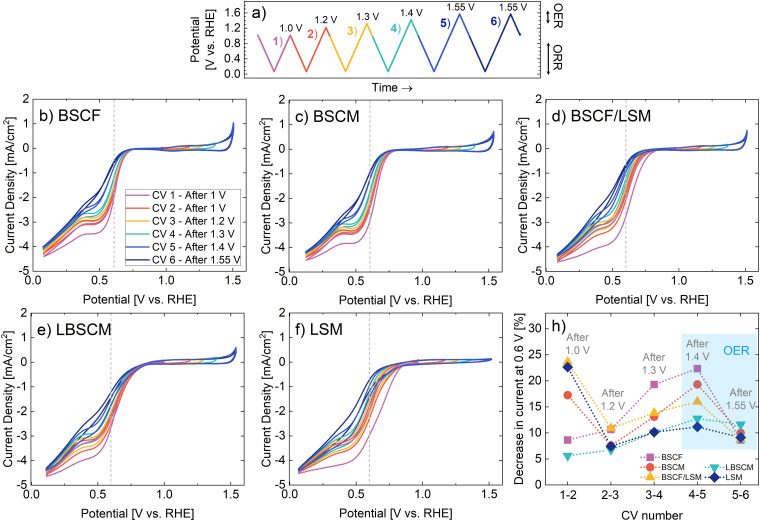
(a) The applied potential with time, which is alternated between reducing and increasing oxidizing potentials. The current densities obtained with RDE for (b) BSCF, (c) BSCM, (d) BSCF/LSM, (e) LBSCM, and (f) LSM in oxygen-saturated 0.1 M KOH with a rotation rate of 1600 rpm and a scan rate of 5 mV s^−1^. (h) The relative percentage of current decrease at 0.6 V between each subsequent CV. The change in current from CV *n* to (*n* + 1) is divided by the initial current of the first CV. The grey dotted lines in (b)–(f) indicate where the current values were taken.

A bifunctional oxygen electrocatalyst must be able to switch reversibly between the oxidizing potentials of the OER and the reducing potentials of the ORR when used in a unified regenerative fuel cell. Alternating operation between the OER and ORR elucidates the performance and stability of the bifunctional catalyst under normal operation in a URFC. Herein, the effect of increasing potentials into the OER region on the ORR activity is investigated. After each CV into the ORR region (1.0–0.05 V), the upper potential limit is increased until OER is reached (1.55 V). The applied potential over time is further described in Fig. 3a and the results for the catalysts are given in Fig. 3(b–f). All catalysts show a shift in the ORR onset to higher overpotentials with each CV. In Fig. 3h the relative percentage of current lost from one CV to the next during the ORR is plotted. The effect of cycling between 1.0 and 0.05 V, without the introduction of oxidizing potentials, is explored in Fig. S5 (ESI†). Within the first three CVs, until 1.2 V, the decrease in ORR activity is due to cycling in ORR conditions rather than the increasing potential into the OER region. From the third CV, the decrease can be attributed to the increasing oxidizing potential limit. BSCF experiences the highest degradation after 1.4 V, where the OER begins to occur. BSCM and BSCF/LSM further exhibit a peak in degradation after 1.4 V. LSM and BSCF/LSM have the largest percentage of performance decrease within the first CV. However, thereafter LSM is not as affected by oxidizing potentials as the other materials. LBSCM has the lowest percentage in current decrease of the studied materials.

Contrarily, the effect of the ORR on the OER activity was also investigated (Fig. S6, ESI†). Interestingly, all the materials that contain both Co and Mn increase in OER activity after oxygen reduction. This is particularly surprising for the composite BSCF/LSM electrode, which has higher OER activity after the ORR even though BSCF and LSM do not. Additionally, for all samples, there is a slight increase in the capacitive current after the ORR.

### 3.3. Time-resolved *operando* XAS

**Fig. 4:**
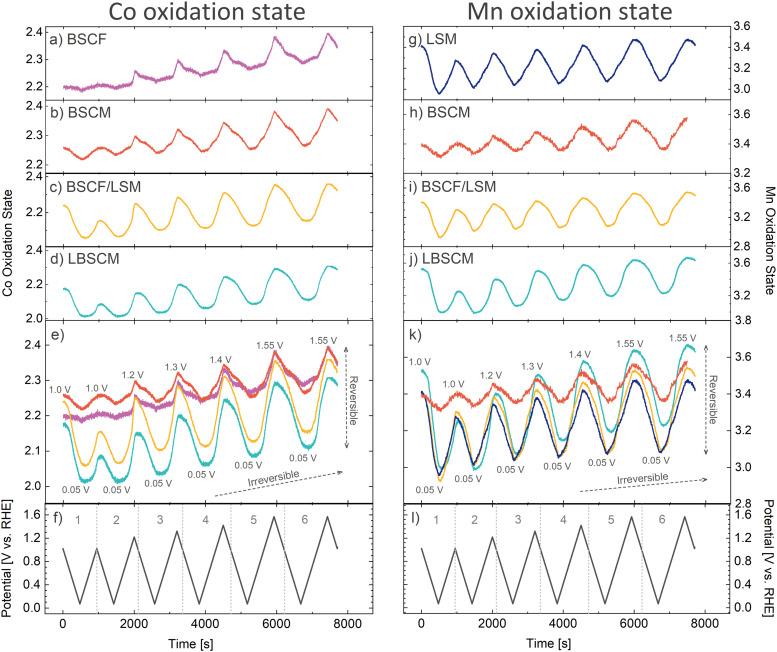
The dynamic changes in the (a)–(e) Co and (g)–(k) Mn oxidation states of the perovskites as the (f) and (l) potential is alternated between reducing and increasingly oxidizing potentials. The oxidation state calculations are explained in Note S1 (ESI†).

The above results have so far demonstrated the influence of the B-site metal and the electrode composition on the oxygen electrocatalysis activity of perovskites. To further elucidate these relationships, *operando* X-ray absorption spectroscopy (XAS) was used to monitor the dynamic changes in oxidation state while cycling between oxidizing and reducing potentials. The same potential opening cyclic voltammetry protocol was performed, as described in Fig. 3a, while XAS was continuously and concurrently measured. The XAS spectra were measured every 0.5 s with a CV scan rate of 2 mV s^−1^. Afterwards, the spectra were averaged for a resolution of one XAS spectrum every 10 mV. The resulting spectra can be analyzed *via* multivariate curve resolution (MCR) and used to estimate the continuous changes in Co and Mn oxidation states as a function of applied potential (Note S1, ESI†). Fig. 4 and Fig. S10 (ESI†) show that with each CV, the oxidation states of Co, Mn, and Fe are reduced during the ORR and oxidized at higher potentials in a cyclic manner, showing reversible and irreversible changes in the oxidation state of the B-site metals during both the ORR and OER.

Within the first CV, where only the ORR occurs, the Co and Mn oxidation states in all samples are reduced when scanning the potential from 1.0 to 0.05 V (Fig. 4). However, there is a stark difference in the extent of Co and Mn oxidation state reduction that occurs. The extent of Co reduction during the ORR for BSCF is minor. However, when Fe (20%) in BSCF is substituted with Mn to create BSCM, the amount of Co reduction increases 3-fold. Contrarily, Mn reduction in BSCM during the ORR is significantly less than the other Mn containing materials, which have roughly the same amount of Mn reduction during the ORR. Even more surprising, is the vastly different behavior of BSCF/LSM compared to BSCF alone. With the physical addition of LSM to BSCF, Co is reduced approximately 9-fold more during the ORR. In contrast, the change in Mn oxidation state for LSM is not significantly altered with the addition of BSCF. The extent of B-site metal reduction during the ORR has previously been correlated with ORR activity in perovskites.^19,32^ This correlation is further verified here; the most ORR active materials (BSCF/LSM, LBSCM, LSM) also have the most significant Co and Mn reduction during the ORR. Although the magnitude of oxidation state change trends with ORR activity, there is no clear trend with OER activity.

After the first CV, the potential window is opened to increasingly oxidizing potentials until the OER is reached in the 5th and 6th CV (Fig. 4). Thereby, it becomes apparent that there is an underlying irreversible oxidation occurring with each cycle, which is particularly strong for Co containing materials. The most likely reason for this is the formation of a Co(Fe)O_*x*_(OH)_*y*_ layer on the surface, which is known to form on BSCF and other Co/Fe containing materials during the OER.^44,69^ This surface layer is predicted to impede the oxygen reduction reaction on BSCF^32^ and would also explain the decrease in ORR activity observed in Fig. 3. The oxyhydroxide layer seems to form on the Co containing materials BSCF, BSCM, BSCF/LSM, and possibly LBSCM during the OER. This is evidenced by the changes in the Fourier-transform extended X-ray absorption fine structure (EXAFS) spectra in Fig. S12 (ESI†). Previously, the formation of a Co oxyhydroxide layer on BSCF and other Co containing perovskites has been predicted during the OER by observing simultaneous changes in the peaks attributable to the Co–Co coordination shells, a growth in the Co–O(OH) peak and a decrease in the peak of the native perovskite.^44,69^ The same trends are observed here during the OER. It seems the formation of the oxyhydroxide layer occurs with and without carbon^19^ and even with LSM added to the catalyst layer. Additionally, these materials experience the irreversible oxidation of Co^2+^ to Co^3+^ after the OER, another potential indicator of oxyhydroxide formation due to the presence of Co^3+^ in CoOOH.^44^ The reducing potentials of the ORR are unable to undo this irreversible oxidation. Therefore, it seems the Co containing materials produce an irreversible self-assembled oxyhydroxide layer during the OER, leading in part to a decrease in ORR activity with cycling between reducing and oxidizing potentials as shown in Fig. 3.

Additionally, a degree of irreversible oxidation can also be observed for Mn. Of materials the studied here, LBSCM has the largest extent of this irreversible oxidation, which may be attributed to its relatively large change in local structure (Fig. S12, ESI†). With each cycle, the ratio of Mn^4+^/Mn^3+^ present during the ORR increases, coordinating with decreased ORR activity, as shown previously in Fig. 3. Mn^3+^ has been predicted to catalyze the first step of the oxygen reduction, the reaction of oxygen to hydrogen peroxide.^73^ In Fig. 4 for BSCF/LSM, LSM, and LSBCM the reduction of Co and Mn during the ORR is much more significant in the first CV compared to the second. This could explain in part the large decrease in ORR activity seen between CV1–2 in Fig. 3 for LSM and BSCF/LSM. However, LBSCM does not show the same degree of performance degradation. Another factor could be the dissolution of Sr that can occur for perovskites such as LSM at ORR potentials.^32^ Sr doping has been shown to lead to improved conductivity, adsorption of oxygen species, and charge-transfer kinetics leading to higher ORR activity in LaMnO_3_.^46,47^ Indeed, under ORR potentials BSCF/LSM, LSM, and LBSCM leach Sr into the electrolyte (Fig. S13, ESI†). However, the dissolution of Sr for LBSCM is significantly less than LSM and BSCF/LSM, possibly explaining the performance trends in Fig. 3h.

An interesting observation is that for the materials which contain both Co and Mn, a physical mixture or single material, the changes in oxidation state are mirrored in both elements indicating a relationship between Co and Mn. Yang *et al.*^74^ found a similar connection between the Co and Mn oxidation states during the ORR for Co_1.5_Mn_1.5_O_4_ and claimed Co and Mn were acting as coactive sites to catalyze the ORR. Additionally, the Fe oxidation state also changes (Fig. S10, ESI†) and roughly mirrors Co, pointing to a possible relationship between Co and Fe as well. Therefore, there seems to be a relationship between the B-site metals during the ORR and OER, which leads to enhanced catalytic activity. The change in oxidation state of the B-site metals appear interconnected and may indicate an interdependency of the B-site metal redox transitions.

**Fig. 5:**
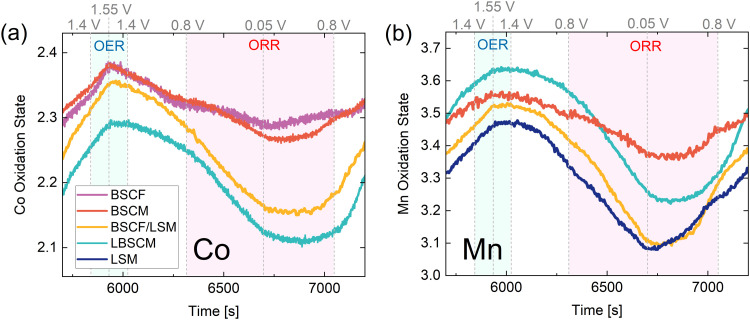
The *operando* XAS results of the 5th and 6th CV (zoomed in region from Fig. 4) at the (a) Co and (b) Mn K-edges. The ORR and OER regions are indicated (blue for OER and red for ORR) to highlight the rate of change of oxidation state in these regions.

Through closer examination of the evolution in the oxidation state over time in Fig. 5, the rates of change of Co and Mn oxidation states can be compared. For all samples, the most reduced and oxidized states do not always occur at the most extreme potentials, which could be an indication of sluggish reaction rates, especially in the ORR region. In particular, as the potential is increased from 0.05 to 0.8 V, most samples do not start to oxidize until much later, indicating slow rates of Co and Mn oxidation on the anodic sweep in the ORR region. Interestingly, for BSCF and LSM the most oxidized and reduced states occur at lower overpotentials than the other materials. During the OER, the slope of Co oxidation state over time experiences a distinct change when switching from an anodic to cathodic sweep, resulting in a sharp peak in Fig. 5a compared to Mn in Fig. 5b.

### 3.4. Anion exchange membrane (AEM) fuel cell and electrolyzer device performance

**Fig. 6:**
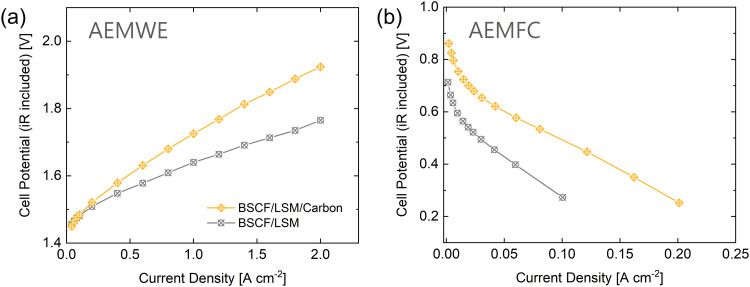
The catalytic performance of BSCF/LSM with and without carbon in AEM devices. (a) AEMWE in 1.0 M KOH at 80 °C. (b) AEMFC with 100% relative humidity at 60 °C. For both measurements, a catalyst loading of 2.0 mg cm^−2^ and a QPAF-4^61^ membrane and ionomer were used. The results are shown *versus* references in Fig. S14 (ESI†).

With the results herein, it becomes clear that the most promising bifunctional catalyst investigated in this study is BSCF/LSM. Therefore, this material was chosen to perform anion exchange membrane (AEM) device characterization to assess the performance of the catalyst in a more applied setting. Fuel cell (AEMFC) and water electrolysis (AEMWE) tests were performed in separate devices. The performance of the catalysts for water electrolysis in Fig. 6a is positive. With only 50 wt% of active OER catalyst (BSCF), BSCF/LSM is able to maintain a low cell voltage under high current densities. As expected, the addition of carbon to the catalyst layer is detrimental to the electrolysis performance, as seen by the higher cell potential of BSCF/LSM/carbon at high current densities. It is predicted that longer stability testing would lead to fast degradation of the BSCF/LSM/carbon catalyst due to carbon corrosion under oxidizing potentials.^75,76^ In contrast, it can be seen that without carbon, the catalyst is unable to achieve high current densities during AEMFC testing most likely due to high ohmic and mass transport voltage losses in Fig. 6b. Carbon increases the conductivity within the catalyst layer as well as the porosity of the catalyst layer, leading to improved gas diffusion. Overall, with the exclusion of carbon from the catalyst layer of BSCF/LSM for URFCs, the fuel cell performance requires improvement in the form of catalyst layer engineering to improve gas diffusion and conductivity.

## 4. Conclusions and recommendations

Two strategies for bifunctional catalyst design have been compared in this work, combining active ORR and OER catalysts into a mixed composite and optimizing a single-phase material for both reactions. The mixed composite BSCF/LSM is able to achieve high OER activity due to a synergistic relationship between the two materials. The addition of LSM to BSCF also leads to enhanced Co reduction during the ORR and increased ORR activity. Overall, BSCF/LSM combines active sites for the ORR and OER without compromising significant activity for either reaction. The attempt to combine active ORR and OER sites within a single-phase material (BSCM and LBSCM) was less successful. Interestingly, the OER was most affected by this combination, leading to a significant loss in OER activity with the addition of Mn and La. The ORR performance did decrease with the substitution of Co and Ba but was not as significantly affected. With the wide range of perovskite materials studied here, it is interesting to note that they all have decreased ORR activity after the OER, while reducing potentials seems to have a more positive effect.

It is questionable whether the apparent synergistic relationship between BSCF and LSM is due to the improved electrical conductivity in the catalyst layer. Even though LSM is more conductive than BSCF by several orders of magnitude, the addition of carbon creates equal conductivity in all the catalyst layers. It is possible that the BSCF particles could still be lacking electronic conductivity locally, which the LSM nanoparticles provide. This would help to explain the surprisingly high OER activity of BSCF/LSM. However, then one would expect a similar performance enhancement to occur for the ORR.

With the results herein, recommendations for future bifunctional catalyst design can be given. Combining a catalyst optimized for the ORR with a catalyst optimized for the OER into a composite seems to be the most promising strategy for bifunctional catalysts. Additionally, this study has shown that the simple process of physically mixing two catalysts together is successful at activating the catalytic activity of both materials and more complex, expensive methods for synthesizing composites are not necessarily required. In the long term, carbon will need to be replaced by an alternative conductive additive, which is stable under OER potentials. It was shown that carbon is essential to achieve high current densities in AEMFC but is detrimental to AEMWE. Regardless, catalyst layer engineering is required to increase the ORR and AEMFC performance. This could include strategies such as morphology changes to increase the surface area, adjusting the A and B site configuration to increase electrical conductivity of the perovskite, or altering the spraying technique to achieve a more porous layer.

## Data availability

The data that support the findings of this study are openly available in Materials Cloud at https://doi.org/10.24435/materialscloud:q7-74.

## Conflicts of interest

The authors declare no conflict of interest.

## Supplementary Material

EY-002-D4EY00084F-s001
