# Supplementary material for: Designing bifunctional perovskite catalysts for the oxygen reduction and evolution reactions
Source: EES Catal. 2024 Jun 24;2(5):1152–63. doi: 10.1039/d4ey00084f (PMC11375951; doi:10.1039/d4ey00084f)
Supplement: EY-002-D4EY00084F-s001 [file EY-002-D4EY00084F-s001.pdf]

Electronic Supplementary Material (ESI) for EES Catalysis.
This journal is © The Royal Society of Chemistry 2024

Supplementary Material

Designing bifunctional perovskite catalysts for the oxygen reduction and evolution reactions

Casey E. Beall[1], Emiliana Fabbri[1*], Adam H. Clark[1], Vivian Meier[1,2], Nur Sena Yüzbasi[3], Thomas Graule[3], Sayaka Takahashi[4], Yuto Shirase[4], Makoto Uchida[4], Thomas J. Schmidt[1,2]

[1]Paul Scherrer Institute (PSI), 5232 Villigen PSI, Switzerland

[2]Institute for Physical Molecular Science, ETH Zürich, 8093 Zürich, Switzerland

[3]Empa, 8600 Dübendorf, Switzerland

[4]Hydrogen and Fuel Cell Nanomaterials Center, University of Yamanashi, 400-0021, Kofu, Japan

*Corresponding Author: Dr. Emiliana Fabbri, emiliana.fabbri@psi.ch

Experimental Methods

Materials Characterization

The electrical conductivity of the perovskite nanopowders were measured using ex situ 4-wire impedance spectroscopy with an amplitude of 500 mV from a frequency of 1 MHz to 10 mHz. The samples were pressed into a pellet under a pressure of 0.6 MPa for 5 minutes. The perovskite/carbon and BSCF/LSM composites were made into a concentrated ink without Nafion (6x more concentrated than the ink used for RDE measurements). The ink was sonicated to create a homogeneous mixture and then dropcasted onto the substrate of the conductivity apparatus. It should be noted that the absolute conductivity values of these measurements should be taken with caution. They are merely meant as a comparison between samples using the same experimental conditions. Brunauer-Emmett-Teller (BET) analysis of N_2 adsorption isotherms (Autosorb-1, Quantachrome Instruments) was performed to find the specific surface area. X-ray diffraction (XRD) was measured on powder samples in Bragg-Brentano mode (Smartlab, Rigaku) with Cu Kα radiation. The transmission electron microscopy (TEM) images were taken by using a cold field emission gun (FEG) at 200 kV as electron beam source (JEM-ARM200F NEOARM, JEOL). Nanopowders were directly deposited on a TEM grid (continuous ultrathin carbon film coated lacey carbon (400 mesh) on copper grid, Sigma-Aldrich) and were stabilized by sputtering carbon on the top (MED010, BalTec). Inductively coupled plasma - optical emission spectrometry (ICP-OES) (5110, Agilent) was performed using axial viewing mode. Electrolyte was collected after 15 CVs (0 - 1.0 V, 10 mV s^{-1}, RDE) and was not diluted. Co, Mn, Fe, Ba, Sr, and La standards were made with concentrations of 10, 1, 0.5, 0.1, and 0.01 ppm.

AEMFC and AEMWE Characterization

An ammonium containing copolymer (QPAF-4) was used for the membrane and binder due to QPAF-4's high hydroxide ion conductivity and excellent mechanical properties and chemical stability.[1] QPAF-4 was synthesized in-house via the procedure outlined by Ono et al.[1] Ion exchange capacities (IEC) of 1.5 (WE) and 2.0 (FC) were used. The membrane thickness was 30 µm (FC) and 50 µm (WE). The membrane and the binder (FC) were ion exchanged by soaking in 1.0 M KOH at 80 °C for 2 days, 0.5 M KCO_3 for one day, and lastly Milli-Q water for one day. Then, dried by pressing between two cloths.

For AEMFC, both anode and cathode inks were made with the following ratio of materials: 0.3 g catalyst, 3.75 g of Milli-Q water, 5.1 g of methanol, and 2.6 g binder solution (5 wt% QPAF-4 in methanol). For AEMWE, the anode ink consisted of the following ratio of materials: 1 g catalyst,

12.6 g of Milli-Q water, 17 g of methanol, and 3 g binder solution (5 wt% QPAF-4 in methanol). The inks were planetary ball milled with 20 zirconia beads (5 mm diameter) for 30 min without the binder solution and a further 30 min with the addition of the filtered QPAF-4-MeOH binder solution. Then, the inks were mixed for at least 12 hours before spraying. The inks were sprayed onto the membrane using a pulse-swirl-spray (PSS, Nordson Co. Ltd.) technique. No pressing or hot-pressing of the MEA was conducted for AEMFC. Low-pressure hot-pressing (0.2 kN, 80 °C) of the MEA occurred for AEMWE.

The samples were tested in an AEMFC set up with an area of 4.41 cm^2 (2.1 cm x 2.1 cm). The cell temperature was 60 °C and the feeding gases were controlled by a mass flow rate controller (100 mL min^{-1}) and humidified to a relative humidity of 100% by bubbling through a hot water reservoir. Silicone/poly(ethyl benzene-1, 4-dicarboxylat)/silicone gaskets (SB50A1P, Maxell Kureha Co., Ltd.) with a thickness of 200 µm and serpentine flow graphite plates were used on both the anode and cathode side and the cell was compressed to a pressure 10 kgf cm^{-2} with four springs. No back pressure was applied to the anode and cathode sides. The anode catalyst consisted of Pt/CB (46.9% Pt, TEC10E50E, Tanaka) with a QPAF-4 binder/carbon ratio of 0.8 and total loading of 0.2 mg_{pt} cm^{-2}. The anode GDL was carbon cloth with MPL (W1S1010, CeTech). The cathode catalyst loading was 2.0 mg cm^{-2} with a QPAF-4 binder/catalyst ratio of 0.43 and the cathode GDL was carbon paper with MPL (22BB, Sigracet). The AEMFC set-up is explained previously in further detail.[2] The current density was increased stepwise until a maximum of 1.0 A cm^{-2} and held at each step for one minute. The cell voltage was monitored as the current density was increased until the limit of approximately 0.2 V was achieved. Then, the current density was subsequently decreased in the same manner. This was repeated until the cell performance no longer increased. The measurement was monitored using an electronic load (PLZ664WA and KFM2150, Kikusui Electronics Corp.) and controlled by a measurement system (fuel cell characteristic evaluation device, Netsuden Kogyo Corp.) The resistance for iR correction was determined from alternating current (AC) impedance (KFM2150, Kikusui Electronics Corp.) for current densities above 0.1 A cm^{-2}. Below this value, resistances are difficult to measure; therefore, a 1 kHz external resistance meter (MODEL 3566, Tsuruga Electric Corp.) was used instead.

For AEMWE measurements, a cell with an area of 1 cm^2 (1 cm x 1 cm) was used. The cell was operated at a temperature of 80 °C and compressed to a pressure of 0.75 MPa. The 1 M KOH electrolyte was preheated to 80 °C and was recirculated with a flow rate of 10 mL min^{-1} after flowing through a 0.6 mm Teflon mesh filter with 5 cm diameter. Approximately 7 L of electrolyte were recirculated and controlled with a mass flow controller (8500MM-0-2-1, Kofloc). EPDM gaskets (Chemix) with 200 µm thickness (anode) and 300 µm thickness (cathode) were employed. The anode GDL was Ni mesh (1Ni06-020, Bekaert) and the cathode GDE was Pt/CB sprayed onto Teflon treated

carbon fiber paper (TGP-H-120, Toray). The anode had a catalyst loading of 2.0 mg cm^{-2}. The anode catalyst layer had a QPAF-4 binder/catalyst ratio of 0.15. The cathode catalyst loading was 1 mg_{pt} cm^{-2} and the QPAF-4 binder/carbon ratio was 0.6. Ni separator plates and a gold plated Cu feeding plate were used. The AEMWE cell was from Chemix by Yokohama National University. The measurement was controlled using a DC power supply (PWR401L, Kikusui) and by a measurement system (electrolyzer characteristic evaluation device, Netsuden Kogyo Corp.). First, the catalyst was activated by increasing the current density twice to 1 A cm^{-2} with steps of 0.2 A cm^{-2}, holding for 30 s at each step, then twice to 2 A cm^{-2}. After activation, a constant current density of 1 A cm^{-2} was applied for 8 hours followed by increasing the current density stepwise to 2 A cm^{-2} twice.

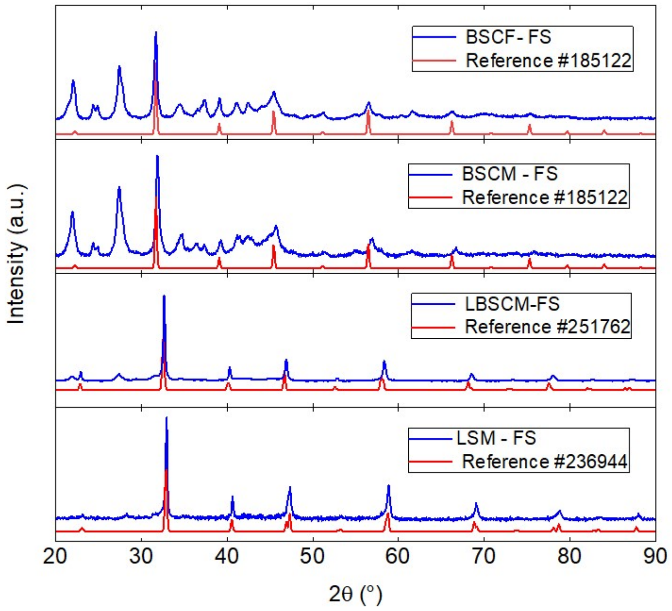

Figure S1. X-ray diffraction patterns of perovskites produced by flame spray synthesis (FS). The patterns are compared to ICSD references. To our knowledge, there are no reported XRD pattern references in the ICSD database that are the same composition as the BSCM and LBSCM synthesized for this study. Therefore, BSCF is used as a reference for BSCM and $La_{0.67}Ba_{0.22}Sr_{0.11}Co_{0.3}Mn_{0.7}O_{3-\delta}$ is used as a reference for LBSCM. The XRD pattern for BSCM shows slightly shifted peaks compared to BSCF, which is expected for a slight change in the B site composition. LBSCM matches closely to the reference of similar composition, also with slightly shifted peaks. The secondary phases present in BSCF and BSCM are mainly Ba and Sr carbonates and nitrates and are explained in the following reference.[3]

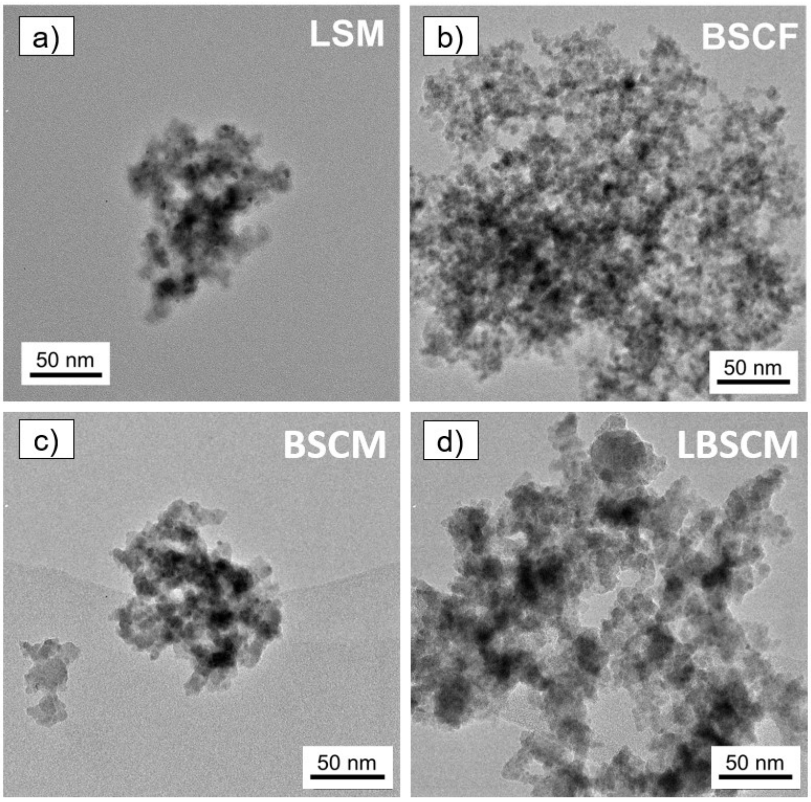

Figure S2. Transmission electron microscopy (TEM) images taken at 200 kV of the nanopowder perovskite samples, a) LSM b) BSCF c) BSCM d) LBSCM, without carbon additives. The nanopowder was lightly deposited on the carbon coated copper grid (400 mesh).

Table S1. Surface area of nanopowder perovskite samples determined using nitrogen adsorption isotherm and Brunauer–Emmett–Teller (BET) theory.

**Sample**	**BET determined surface area (m^2/g)**
BSCF	6 ± 0.5
BSCM	6 ± 0.5
LBSCM	4 ± 0.5
LSM	12 ± 0.5

Table S2. Results from fitting the Nyquist plots obtained from impedance spectroscopy measurements of nanopowder perovskite samples without carbon.

Sample	Conductivity (S cm^{-1})
BSCF	2.2×10^{-13}
BSCM	2.2×10^{-12}
LBSCM	1.3×10^{-8}
LSM	8.5×10^{-10}

Table S3. Results obtained from impedance spectroscopy measurements of the composites perovskite/carbon and BSCF/LSM. In order to obtain a homogeneous catalyst layer, a concentrated ink (without Nafion) containing a mixture of materials was sonicated and then dropcasted onto the substrate of the 4-wire conductivity apparatus.

Sample	Conductivity (S cm^{-1})
BSCF/Carbon	1.7×10^{-4}
BSCM/Carbon	2.2×10^{-4}
LBSCM/Carbon	1.3×10^{-4}
LSM/Carbon	2.0×10^{-4}
BSCF/LSM/Carbon	1.9×10^{-4}
BSCF/LSM	2.5×10^{-10}

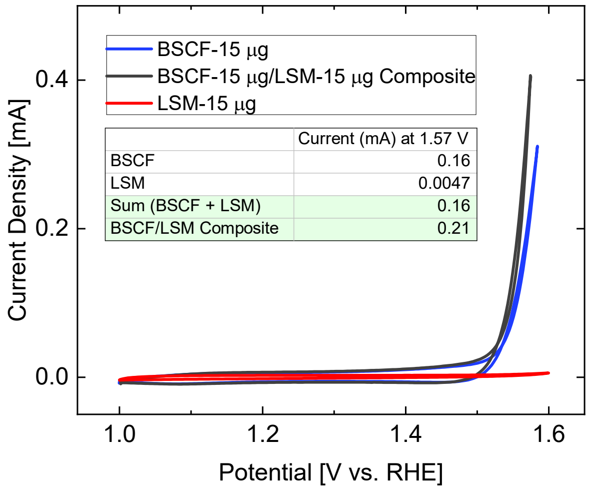

Figure S3. In order to determine the influence of mixing BSCF and LSM together in the catalyst layer on OER activity, BSCF and LSM loading experiments using RDE in oxygen saturated electrolyte were measured. Three CVs were applied between 1.0 - 1.6 V and the third CV is shown here. Lower loadings were used in order to be certain of full catalyst utilization. Then, the same amount of BSCF and LSM that were added into the mixed composite (BSCF/LSM) were measured separately. The composite contained 15 μg of BSCF and 15 μg of LSM deposited onto the glassy carbon surface. Therefore, a separate RDE measurement of 15 μg of BSCF was evaluated for OER activity and subsequently another measurement of 15 μg of LSM. Afterwards, the OER activity (current measured at 1.57 V on the anodic sweep) of the separate BSCF and LSM measurements were added together and compared to the OER activity of the composite BSCF/LSM. The mixed composite is more active for OER than the sum of its counterparts so there is most likely a relationship between BSCF and LSM that is leading to higher OER activity.

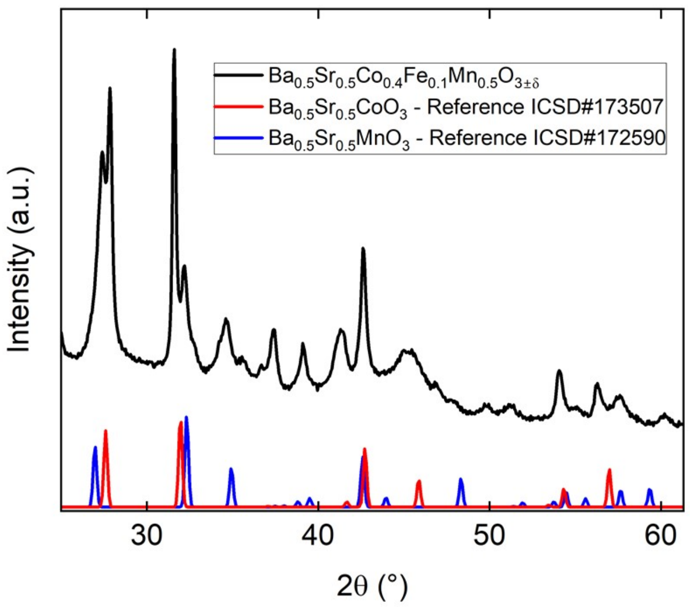

Figure S4. The X-ray diffraction pattern of the perovskite $Ba_{0.5}Sr_{0.5}Co_{0.4}Fe_{0.1}Mn_{0.5}O_{3-\delta}$ produced with flame-spray synthesis. The results are compared to references from the ICSD database. The produced sample most likely contains two perovskites $Ba_{0.5}Sr_{0.5}CoO_{3-\delta}$ and $Ba_{0.5}Sr_{0.5}MnO_{3-\delta}$ instead of one coherent perovskite structure.

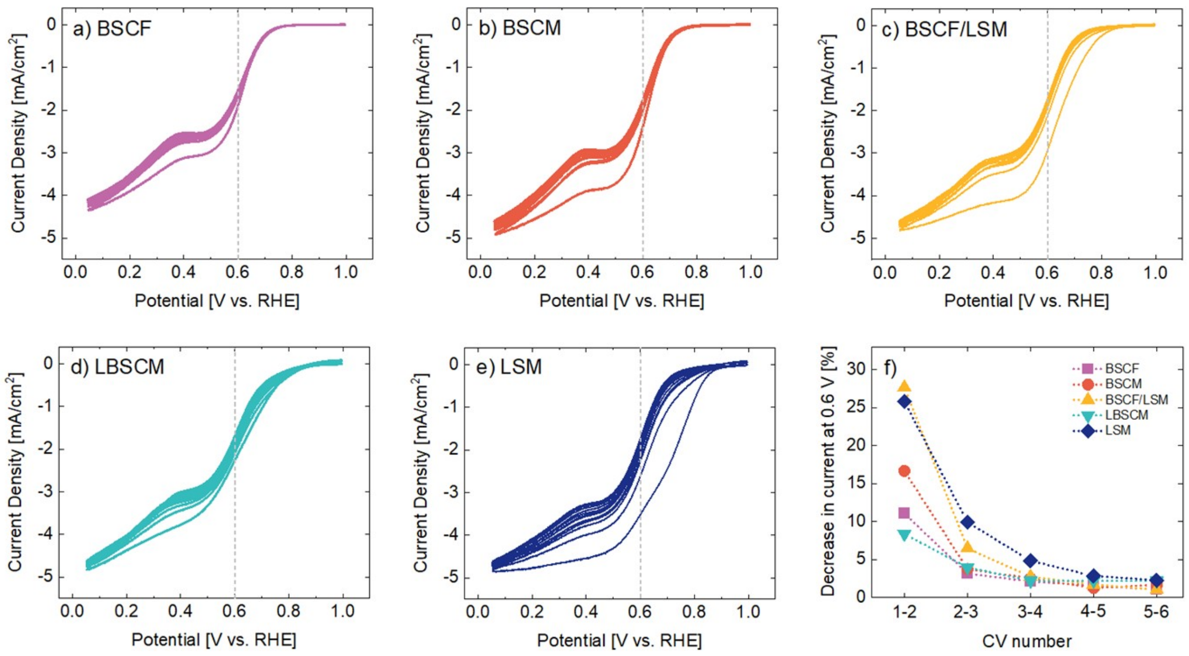

Figure S5. Cycling in the ORR region using RDE. Six CVs between 0.0 – 1.0 V for a) BSCF b) BSCM c) BSCF/LSM d) LBSCM and e) LSM. f) The relative percentage of current decrease at 0.6 V between each subsequent CV. The change in current from CV n to $(n+1)$ is divided by the initial current of the first CV. The grey dotted lines in a-e indicate where the current values were taken.

Note S1: – The oxidation states were estimated by taking the energy at 0.5 normalized intensity of the adsorption onset of the XANES spectra and comparing them to references. These energy values of the samples lie in between those of the references and therefore, one can extrapolate which oxidation state the sample has. For the Co oxidation states, the spectra were compared to $Co(OH)_2$ and CoOOH. For Mn, the references were MnO, Mn_2O_3, MnO_2. For Fe, the references are Fe_2O_3 and Fe_3O_4. Additionally, 0.75 normalized intensity was used for the Co K-edge because BSCF/LSM has a pre-edge feature around 0.5 which leads to artifically low oxidation states. To determine the fluxuating oxidation states during electrochemistry, the MCR concentrations were multiplied by the extrapolated oxidation states.

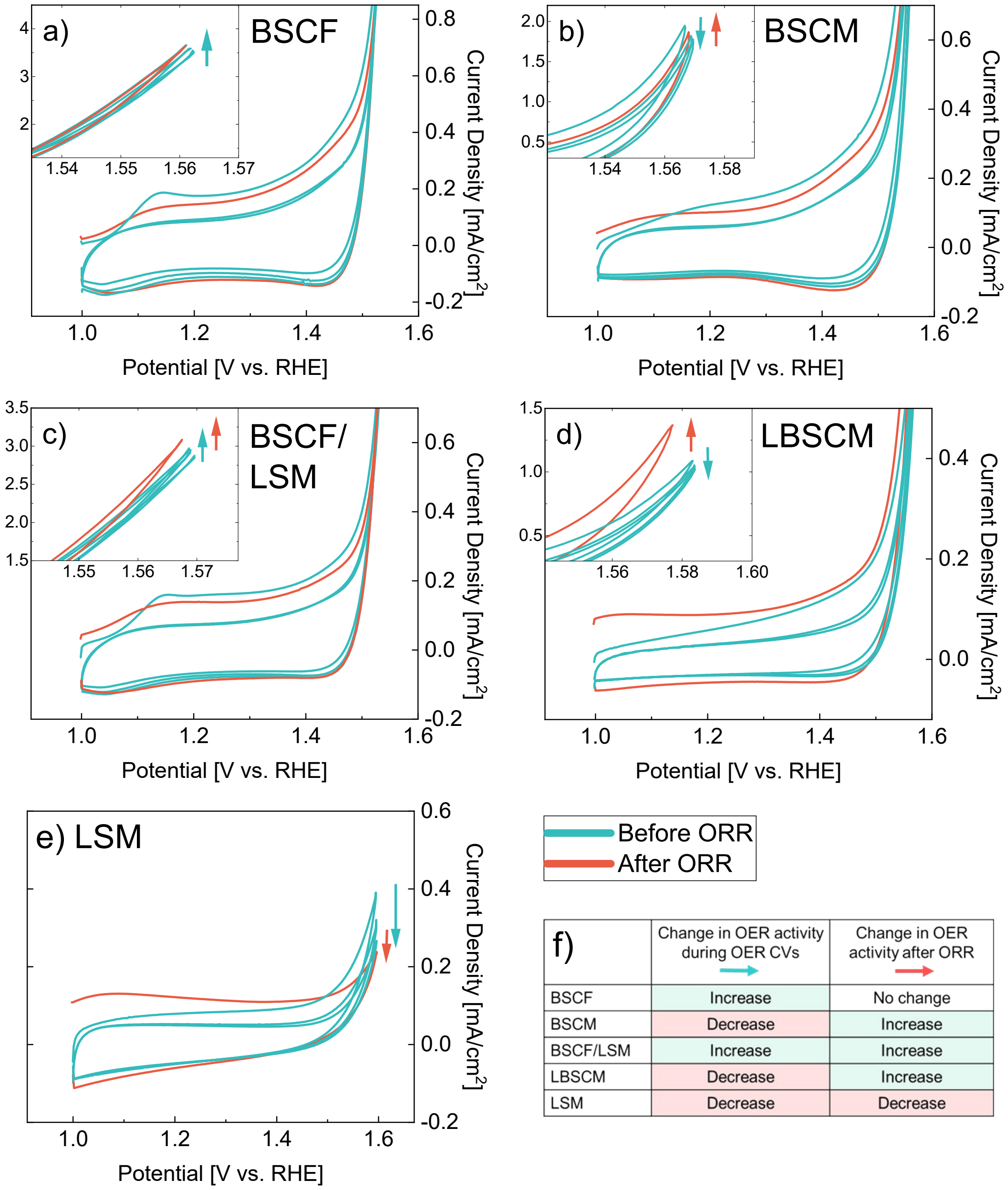

f)	Change in OER activity during OER CVs	Change in OER activity after ORR
BSCF	Increase	No change
BSCM	Decrease	Increase
BSCF/LSM	Increase	Increase
LBSCM	Decrease	Increase
LSM	Decrease	Decrease

Figure S6. The effect of ORR potentials on the OER activity measured with RDE. First, three OER CVs (1.0 - 1.6 V) were measured and the change in OER activity with time was noted. Then, one ORR CV (1.0 – 0.0 V) was performed. Lastly, one OER CV was performed to investigate the change in OER activity after ORR. The results are shown for a) BSCF, b) BSCM, c) BSCF/LSM, d) LBSCM, and e) LSM and summarized in f).

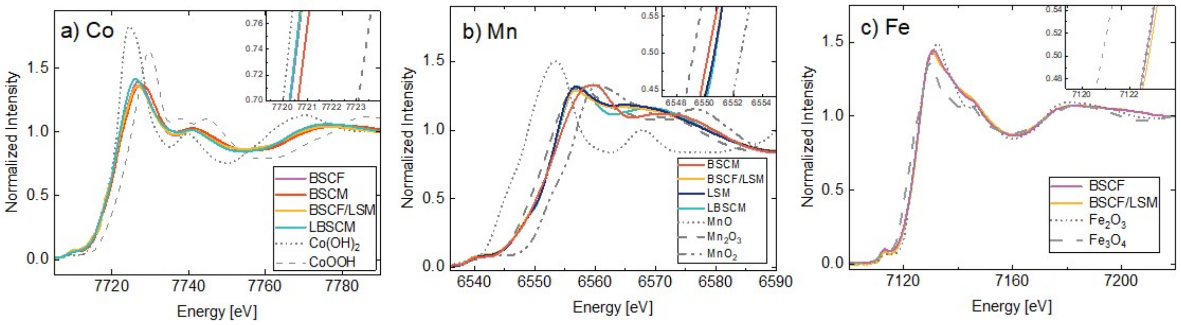

Figure S7. The initial oxidation states of the perovskite samples compared to key oxidation state references at the a) Co, b) Mn, and c) Fe K-edges.

Table S4. Initial oxidation states determined by comparing dry samples to reference samples shown in **Figure S7**.

	Co Oxidation State	Fe Oxidation State	Mn Oxidation State
BSCF	2.1	3.0	N/A
BSCM	2.2	N/A	3.3
BSCF/LSM	2.1	3.0	3.4
LBSCM	2.1	N/A	3.5
LSM	N/A	N/A	3.4

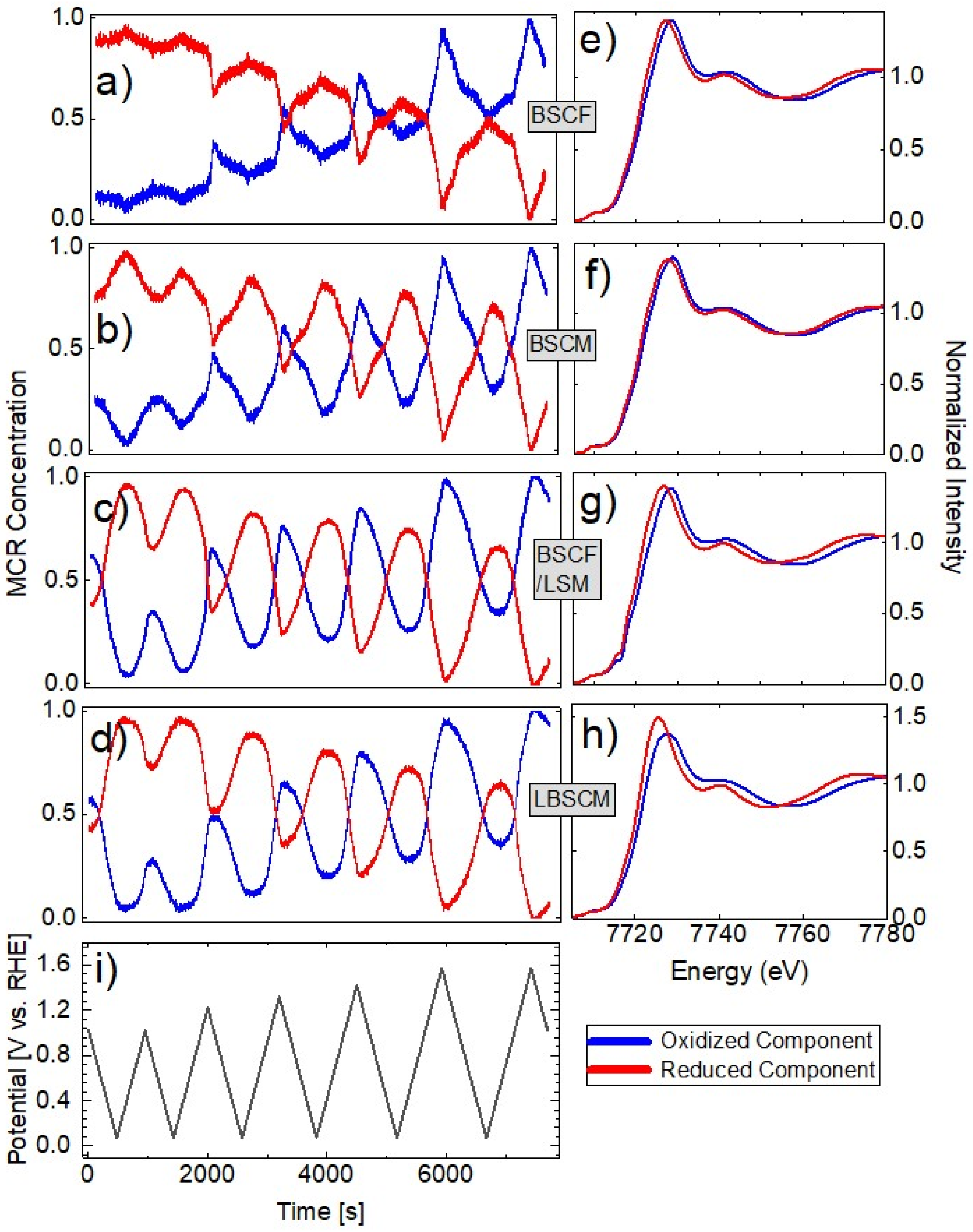

Figure S8. The MCR results at the Co K-edge for Co containing samples. The MCR concentrations for the oxidized and reduced components are shown in a)-d). The resulting spectra for these components are shown to the right in e)-h). The corresponding applied potential vs. time is shown in i).

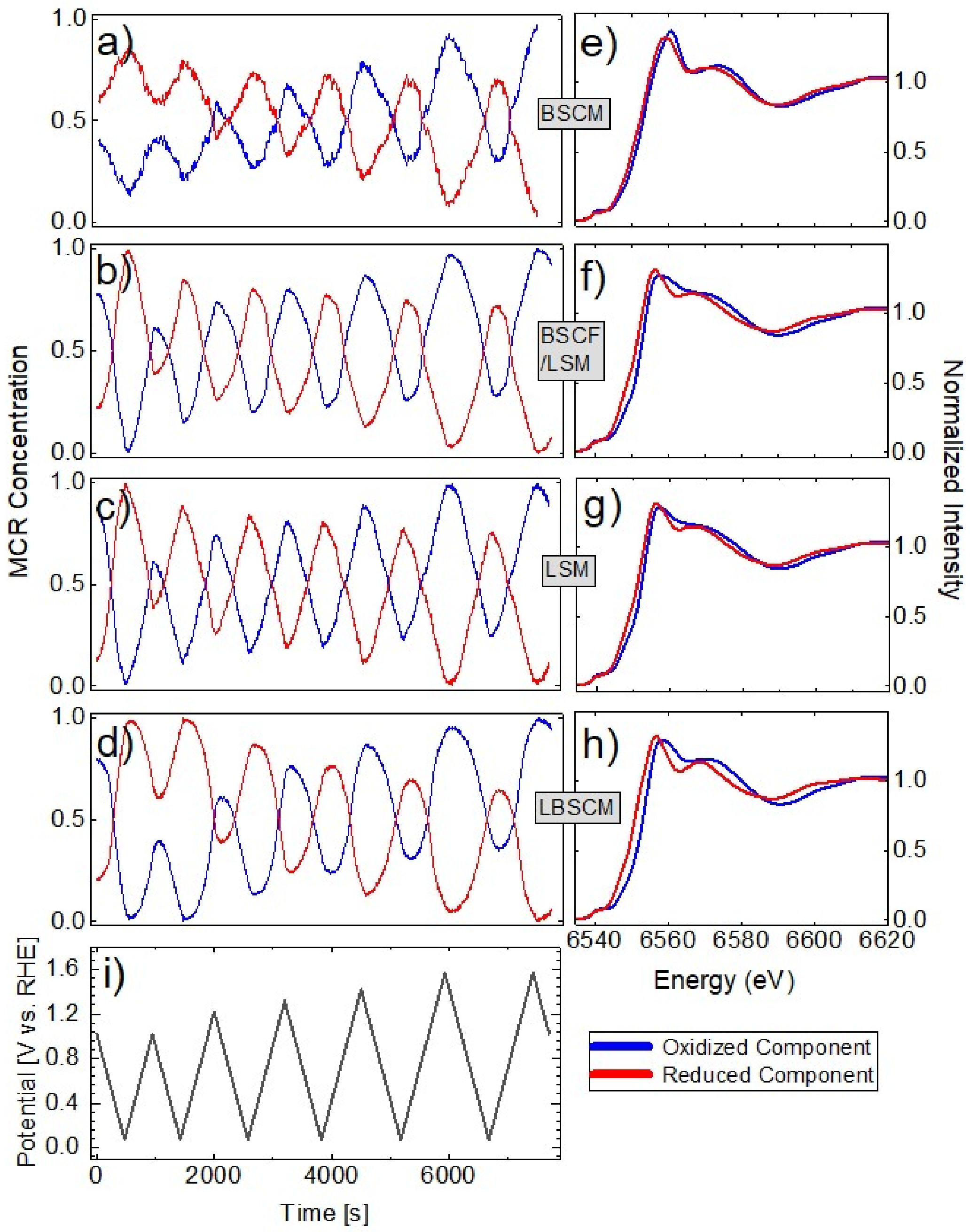

Figure S9. The MCR results at the Mn K-edge for Mn containing samples. The MCR concentrations for the oxidized and reduced components are shown in a)-d). The resulting spectra for these components are shown to the right in e)-h). The corresponding applied potential vs. time is shown in i).

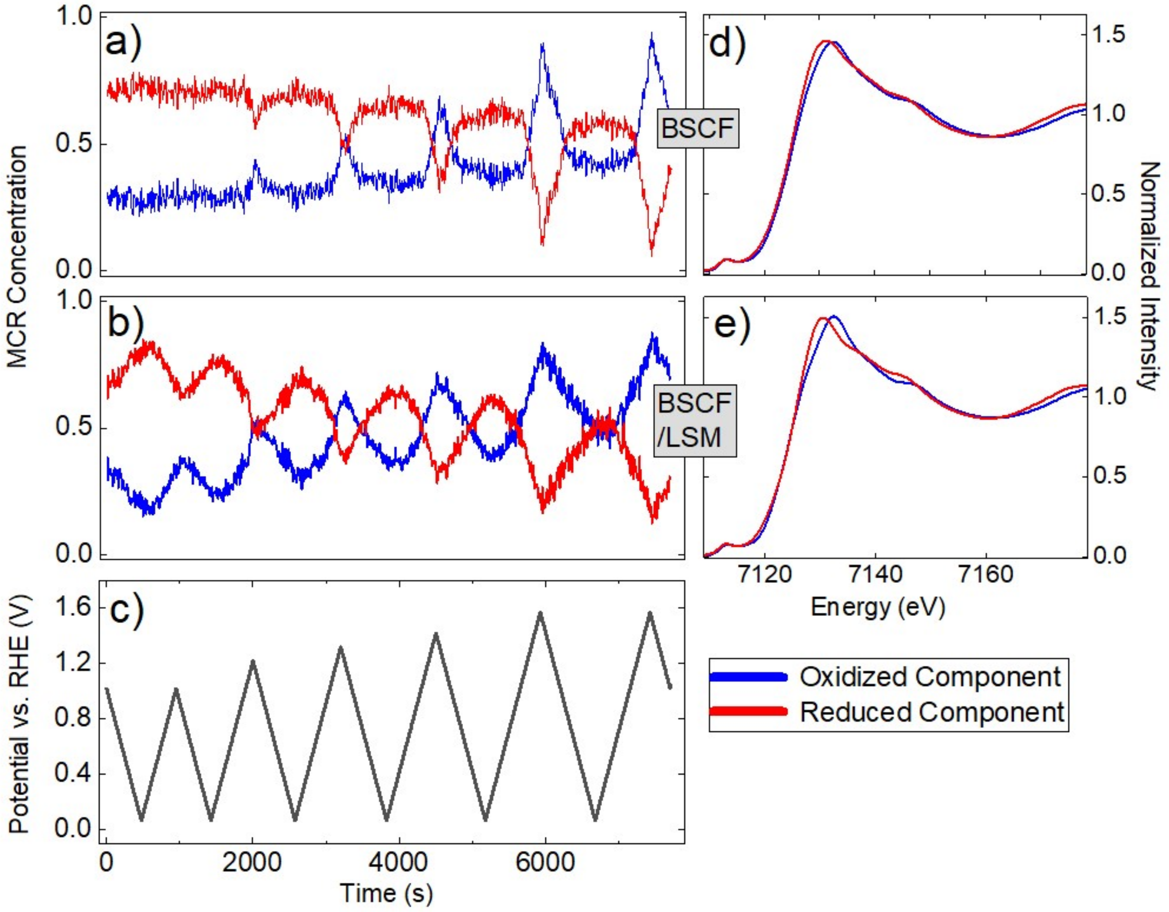

Figure S10. The MCR results at the Fe K-edge for Fe containing samples. The MCR concentrations for the oxidized and reduced components are shown in a,b). The resulting spectra for these components are shown to the right in d,e). The corresponding applied potential vs. time is shown in c).

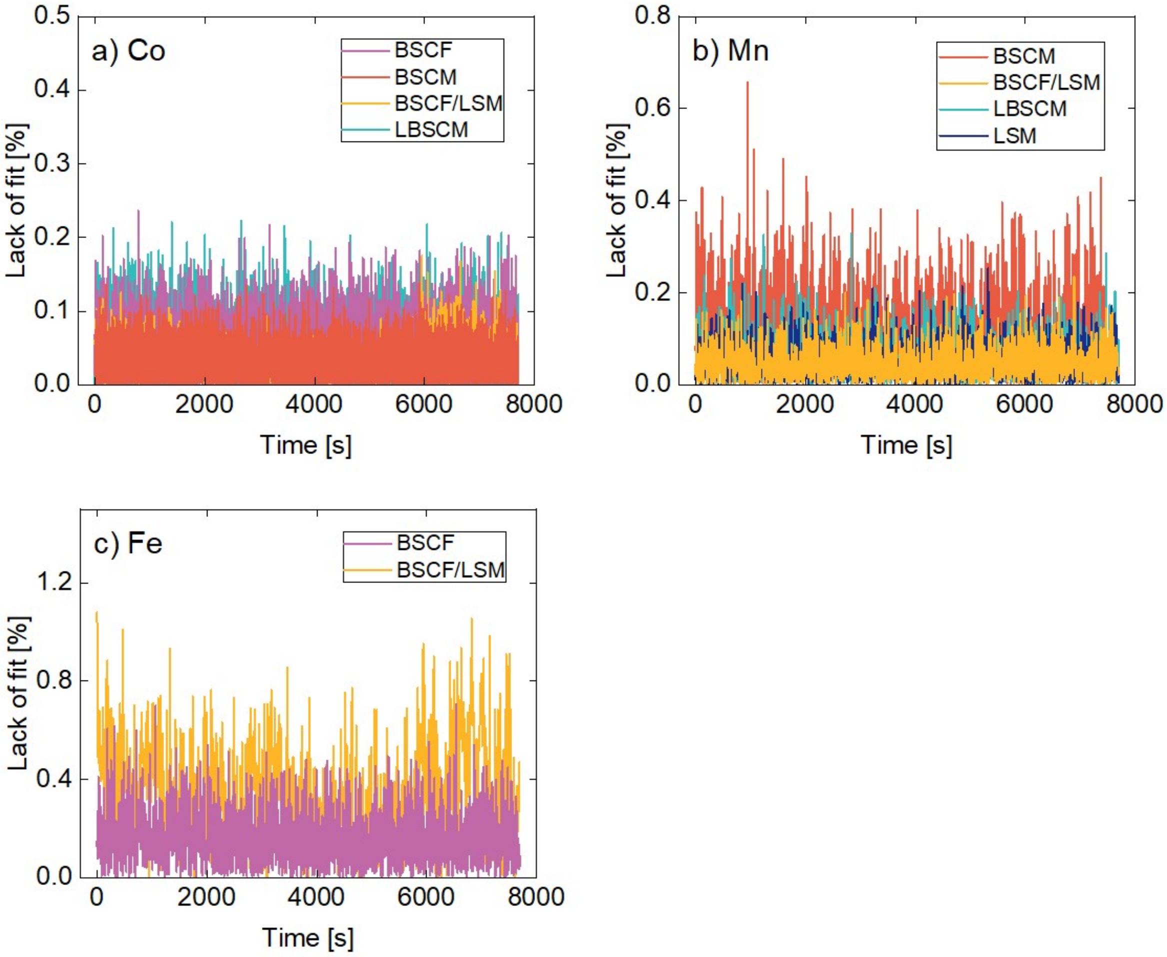

Figure S11. The lack of fit from the MCR analysis of the operando XAS data shown in **Figure S8**, **Figure S9**, and **Figure S10**.

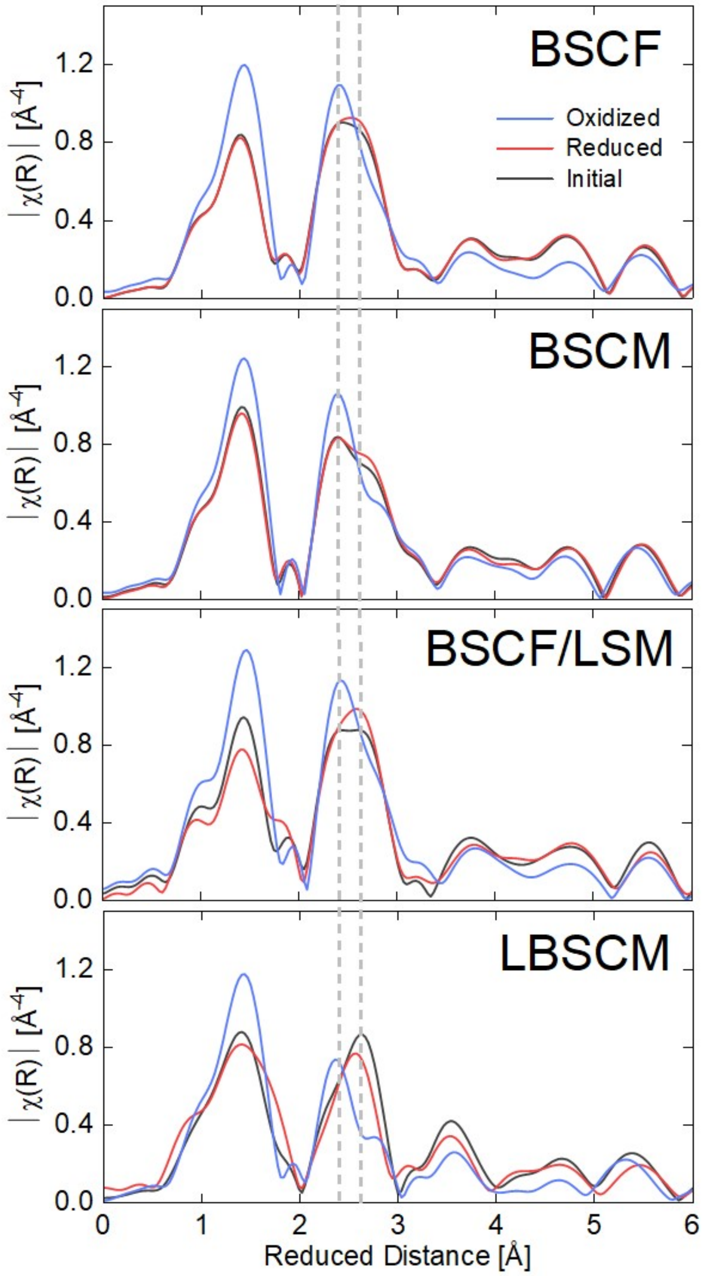

Figure S12. The Fourier-transformed extended X-ray absorption fine structure (EXAFS) spectra of the perovskite materials during operando XAS measurements. The same protocol as described in Figure 4 was used. The two most divergent states, one oxidized and one reduced, that occurred throughout the protocol, as determined from MCR analysis, are shown compared to the initial state. The ProQEXAFS software was used for data analysis, energy calibration, and normalization.[4] The EXAFS spectra were analyzed with the Demeter software package.[5] The spectra were Fourier-transformed over 3 – 12 Å and k^2-weighted. The growth of a Co oxyhydroxide layer on the surface of BSCF is suggested by the decrease intensity of the peak around 2.6 Å, which can be attributed to Co^{2+} occupying the perovskite lattice, and the simultaneous increase in the peak around 2.4 Å, attributable to Co^{3+} edge-sharing octahedral sites in a CoOOH structure.[6,7] This change is apparent for all samples when comparing the initial state to the oxidized state (OER).

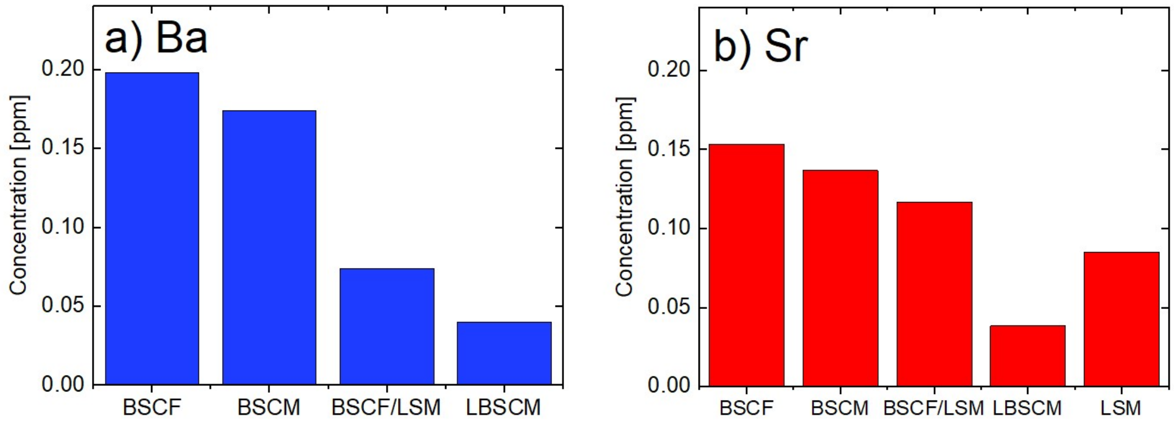

Figure S13. Elemental concentrations of a) Ba and b) Sr detected using ICP-OES after ORR. ORR was performed with RDE (15 CVs 0-1 V at 10 mV s-1) and the electrolyte was collected afterwards for ICP analysis. Other elements Co, Fe, Mn, and La were not detected in significant enough amounts to be identified.

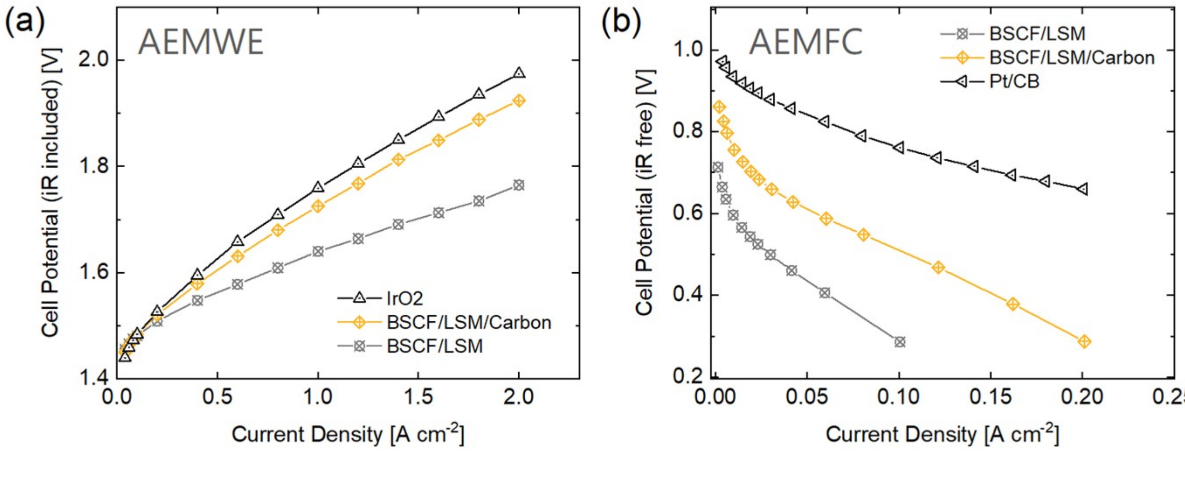

Figure S14. The catalytic performance of BSCF/LSM with and without carbon in AEM devices. a) AEMWE in 1.0 M KOH at 80 °C. b) AEMFC with 100% relative humidity at 60 °C. For both measurements, a catalyst loading of 2.0 mg cm^{-2} and a QPAF-4 membrane and ionomer were used. The results are shown versus industry standards IrO_2 and Pt/CB catalysts. However, it should be noted that these are standards for electrolyzer and fuel cell, respectively and not for URFC devices. Therefore, the comparison of Pt/CB and IrO_2 versus BSCF/LSM is not equal. BSCF/LSM must be active for both reactions while Pt/CB is optimized only for ORR and IrO_2 only for OER.

References

(1) Ono, H.; Kimura, T.; Takano, A.; Asazawa, K.; Miyake, J.; Inukai, J.; Miyatake, K. Robust Anion Conductive Polymers Containing Perfluoroalkylene and Pendant Ammonium Groups for High Performance Fuel Cells. *J. Mater. Chem. A* **2017**, *5* (47), 24804–24812. https://doi.org/10.1039/c7ta09409d.

(2) Otsuji, K.; Shirase, Y.; Asakawa, T.; Yokota, N.; Nagase, K.; Xu, W.; Song, P.; Wang, S.; Tryk, D. A.; Kakinuma, K.; Inukai, J.; Miyatake, K.; Uchida, M. Effect of Water Management in Membrane and Cathode Catalyst Layers on Suppressing the Performance Hysteresis Phenomenon in Anion-Exchange Membrane Fuel Cells. *J. Power Sources* **2022**, *522*, 230997. https://doi.org/10.1016/J.JPOWSOUR.2022.230997.

(3) Aegerter, D.; Borlaf, M.; Fabbri, E.; Clark, A. H.; Nachtegaal, M.; Graule, T.; Schmidt, T. J. Tuning the Co Oxidation State in Ba0.5Sr0.5Co0.8Fe0.2O3-δ by Flame Spray Synthesis Towards High Oxygen Evolution Reaction Activity. *Catalysts* **2020**, *10* (9), 984. https://doi.org/10.3390/catal10090984.

(4) Clark, A. H.; Imbao, J.; Frahm, R.; Nachtegaal, M. ProQEXAFS : A Highly Optimized Parallelized Rapid Processing Software for QEXAFS Data. *J. Synchrotron Radiat.* **2020**, *27* (2), 551–557. https://doi.org/10.1107/S1600577519017053.

(5) Ravel, B.; Newville, M. ATHENA, ARTEMIS, HEPHAESTUS: Data Analysis for X-Ray Absorption Spectroscopy Using IFEFFIT. In *Journal of Synchrotron Radiation*; International Union of Crystallography, 2005; Vol. 12, pp 537–541. https://doi.org/10.1107/S0909049505012719.

(6) Fabbri, E.; Nachtegaal, M.; Binninger, T.; Cheng, X.; Kim, B.-J.; Durst, J.; Bozza, F.; Graule, T.; Schäublin, R.; Wiles, L.; Pertoso, M.; Danilovic, N.; Ayers, K. E.; Schmidt, T. J. Dynamic Surface Self-Reconstruction Is the Key of Highly Active Perovskite Nano-Electrocatalysts for Water Splitting. *Nat. Mater.* **2017**, *16* (9), 925–931. https://doi.org/10.1038/nmat4938.

(7) Kim, B.-J.; Fabbri, E.; Abbott, D. F.; Cheng, X.; Clark, A. H.; Nachtegaal, M.; Borlaf, M.; Castelli, I. E.; Graule, T.; Schmidt, T. J. Functional Role of Fe-Doping in Co-Based Perovskite Oxide Catalysts for Oxygen Evolution Reaction. *J. Am. Chem. Soc.* **2019**, *141* (13), 5231–5240. https://doi.org/10.1021/jacs.8b12101.
